# Genetic Structure Analyses of Red Imported Fire Ants in Zhejiang Reveal Multiple Introduction Sources in Eastern China

**DOI:** 10.3390/insects17060597

**Published:** 2026-06-07

**Authors:** Xinzhi Hu, Rui Liu, Xinyi Yang, Wenzheng Guo, Kaihui He, Meihong Ni, Mingxing Jiang

**Affiliations:** Ministry of Agriculture and Rural Affairs Key Laboratory of Molecular Biology of Crop Pathogens and Insect Pests, Zhejiang Key Laboratory of Biology and Ecological Regulation of Crop Pathogens and Insects, Zhejiang Engineering Research Center for Biological Control of Crop Pathogens and Insect Pests, Institute of Insect Sciences, Zhejiang University, Hangzhou 310058, China; xinzhi_hu@zju.edu.cn (X.H.); 19966112566@163.com (R.L.); xyyang1026@163.com (X.Y.); 13867133214@163.com (W.G.); 18224615800@163.com (K.H.)

**Keywords:** *Solenopsis invicta*, genetic structure, microsatellites, invasive ant

## Abstract

Red imported fire ants (*Solenopsis invicta*) have invaded Zhejiang Province in eastern China, but their introduction sources remain unclear. Using 42 microsatellite markers, we analyzed the genetic structure of 116 ant colonies from 35 locations across Zhejiang and seven other Chinese provinces. Our results reveal multiple, genetically distinct clusters within Zhejiang. The Ningbo population is highly differentiated from all other sampled Chinese populations, suggesting a possible overseas origin (e.g., via port entry), a hypothesis not previously considered. Despite geographical proximity, the Jinhua and Dongyang populations are genetically distinct, indicating separate sources: Jinhua from southern China via nursery stock transport, and Dongyang from imported logs. Populations in Wenzhou, Jiaxing and Hangzhou are genetically closer to samples from other Chinese provinces than to other Zhejiang populations. These findings demonstrate that *S. invicta* in Zhejiang originated from multiple independent introduction events, including both domestic and international sources. This complex genetic structure highlights the need for strengthened quarantine measures at ports (especially Ningbo) and reduced human-mediated dispersal within the province to prevent admixture that could enhance invasive potential. However, we acknowledge that the limited sample sizes (mostly three nests per population) may affect the precision of allele frequency-based estimates, and our findings should be interpreted with this caveat.

## Simple Summary

Red imported fire ants are one of the world’s most destructive invasive species. In China, they have spread to more than 700 counties since they were first detected in 2004. Zhejiang Province, in eastern China, first found these ants in 2016, and they have since spread widely. To manage and control this pest effectively, it is essential to understand where the ants in Zhejiang originally came from. In this study, we used a DNA-based method (microsatellite markers) to examine the genetic makeup of fire ant populations across Zhejiang and a number of other Chinese provinces. Our results revealed that fire ants in Zhejiang have arrived from multiple different sources. For instance, ants in Ningbo likely came from overseas via port entry, a possibility not previously recognized; ants in Jinhua probably originated from southern China through the movement of nursery plants, while ants in Dongyang likely arrived with imported logs. Furthermore, populations in Wenzhou, Jiaxing and Hangzhou are genetically more similar to those in other Chinese provinces than to other Zhejiang populations. These findings indicate that Zhejiang has been invaded by fire ants multiple times through independent events, involving both domestic and international sources. Understanding this complexity helps guide quarantine and management efforts, such as strengthening inspections at ports (especially Ningbo) and reducing the transport of infested nursery plants between regions, to prevent further spread and the mixing of genetically distinct populations that could make the ants even harder to control.

## 1. Introduction

Invasions by insects, including various ant species, are escalating globally, driven by human activities, climate change, and the increasing interconnectedness of the world [1,2]. These invasions pose direct or indirect impacts on ecosystems, economic growth, and human health [3,4,5]. To prevent the introduction and spread of invasive ants, researchers have adopted various methods to trace their invasion sources and dispersal pathways, including the use of genetic markers such as cytochrome c oxidase subunit I (*COI*) sequencing and microsatellite genotyping (e.g., [6,7]). Microsatellites have proven to be effective tools in this field (e.g., [8,9]) and have also been applied to the red imported fire ant, *Solenopsis invicta*, in several studies [10,11,12,13,14,15,16].

*S. invicta* is listed as one of the world’s 100 most destructive invasive species [17]. Native to the Paraná River basin in South America, it has now been detected in approximately 20 countries and regions across the Americas, Asia, Oceania, and Europe outside its native range [12,18,19]. In mainland China, this ant is spreading rapidly [20]. Since its first detection in 2004, it has invaded more than 700 counties across 13 provinces (data updated to 30 June 2025; [21]). Global warming and human activities are expected to further promote its expansion into more northern regions [22,23,24]. Wuchuan in Guangdong Province, southern China, has been suggested as the source of subsequent invasions within the country [14], and long-distance transport to other provinces is largely driven by human activities [25,26].

Zhejiang Province, located in eastern China, first detected *S. invicta* in 2016, and the ant has since spread widely across the province. Several recent studies have investigated the ecology of *S. invicta* in Zhejiang, including nest-relocation behavior [27], inter-nest colony connections [28], and factors potentially inhibiting its spread such as native ants [29] and landscape features [30]. More recently, based on *COI* haplotypes, Huang et al. [31] showed that fire ants in Zhejiang were originally introduced from Guangdong (the earliest invaded province in mainland China) and also from Fujian (neighboring southern Zhejiang), likely via the transport of horticultural plants. However, the introduction sources and pathways in Zhejiang remain far from fully understood. First, fire ant populations that may have an overseas origin have not been considered. For example, large quantities of logs (used for furniture production) are imported from other countries to several towns in Zhejiang, along which fire ants could be introduced. Second, regarding fire ants believed to be transported with nursery stocks, it is unclear whether they could also originate from provinces other than Guangdong [31]. Given the dense transportation network in Zhejiang, nursery stocks entering the province likely come from multiple sources rather than a single region (e.g., Guangdong). Third, in addition to imported logs and nursery stocks, other materials such as waste plastics, turf, or scrap leather may also carry fire ants and serve as introduction sources [32]. Therefore, the introduction sources of *S. invicta* in Zhejiang may be more complex than previously speculated and require further clarification.

Understanding the introduction sources of fire ants in Zhejiang will help evaluate their future spread tendency in eastern China. If fire ants in this province originate from different sources, they may acquire high levels of genetic diversity through population admixture. Consequently, this could enhance their ability to adapt to new environments and spread into regions that would otherwise be unsuitable for establishment. Moreover, such knowledge will also help assess the significance of bridgehead effects (a factor potentially contributing to global spread of invasive ants [33]) for future fire ant spread. For instance, Jinhua, located in central Zhejiang and serving as a nursery center for horticultural saplings, may become a bridgehead for further invasions due to heavy fire ant infestations and frequent transport of saplings to other regions. Thus, information on the genetic structure and invasion sources in Jinhua would assist in analyzing the sources of fire ants in newly invaded regions such as Shanghai and Jiangsu, and also provide guidance for developing control strategies.

**Figure 1:**
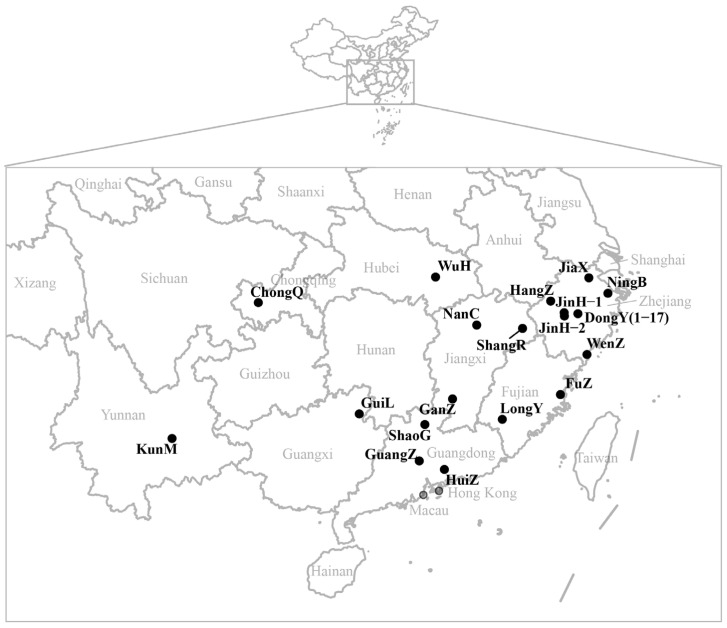
Sampling locations of *Solenopsis invicta* in this study. Black dots indicate sampling sites. Population IDs correspond to those listed in Table 1. DongY (1–17) stands for populations numbered from 1 to 17 collected from Dongyang, Zhejiang.

In this study, using dozens of microsatellite loci as markers, we examined the genetic structure of *S. invicta* populations in Zhejiang, as well as populations collected from other Chinese provinces (Figure 1). Based on the identified genetic relationships among populations, we inferred the introduction sources of *S. invicta* in Zhejiang. Among the populations collected in Zhejiang, some were specifically sampled from Dongyang, which imports logs from Southeast Asia, Africa, and Europe, and from Jinhua, the aforementioned nursery center of horticultural saplings. We also included populations from locations distant from Dongyang and Jinhua, covering different parts of Zhejiang. Our hypothesis is that fire ants occurring in Zhejiang have multiple introduction sources, which can be revealed by the genetic relationships among geographic populations. The aims of this study are to uncover the current genetic characteristics of *S. invicta* in Zhejiang, ascertain the possibility of multiple invasion sources, and confirm the need to prevent fire ant populations in Zhejiang from increasing their genetic diversity through admixture.

**Table 1:** Sampling information of *Solenopsis invicta* worker ants in this study.

Province/Region	Collection Locality	Population No.	Population ID	Nests Sampled (*N*)	GPS Coordinate (Latitude, Longitude)
Zhejiang	Dongyang	1	DongY-1	3	29.09–29.15° N, 120.19–120.24° E
		2	ChangF	3	
		3	GeF	3	
		4	DongJH	3	
		5	JiuN	3	
		6	JinS	3	
		7	JiuW	3	
		8	JiuWN	3	
		9	JunY	3	
		10	LiG	3	
		11	QuanF	3	
		12	QianJ	3	
		13	QianZ	3	
		14	ShangZ	2	
		15	XiaoX	3	
		16	YuF	3	
		17	ZhiS	4	
	Jinhua	18	JinH-1	3	29.16° N, 119.62° E
		19	JinH-2	20	29.03° N, 119.63° E
	Hangzhou	20	HangZ	3	29.61° N, 119.04° E
	Wenzhou	21	WenZ	3	27.54° N, 120.60° E
	Jiaxing	22	JiaX	3	30.51° N, 120.68° E
	Ningbo	23	NingB	1	29.91° N, 121.50° E
Jiangxi	Nanchang	24	NanC	3	28.68° N, 115.86° E
	Shangrao	25	ShangR	3	28.55° N, 117.83° E
	Ganzhou	26	GanZ	3	25.82° N, 114.82° E
Fujian	Fuzhou	27	FuZ	3	25.99° N, 119.46° E
	Longyan	28	LongY	1	25.03° N, 116.96° E
Guangdong	Guangzhou	29	GuangZ	3	23.42° N, 113.39° E
	Huizhou	30	HuiZ	1	23.09° N, 114.47° E
	Shaoguan	31	ShaoG	3	24.83° N, 113.63° E
Hubei	Wuhan	32	WuH	3	30.54° N, 114.09° E
Guangxi	Guilin	33	GuiL	3	25.24° N, 110.81° E
Yunnan	Kunming	34	KunM	3	24.29° N, 102.76° E
Chongqing	Chongqing	35	ChongQ	3	29.55° N, 106.47° E

## 2. Materials and Methods

### 2.1. Ant Collection

A total of 116 *S. invicta* samples, each derived from a single nest, were collected from 35 locations of eight provinces in China. These samples were grouped into 35 populations (Table 1). Among them, 23 populations were from Zhejiang province, including: (1) 17 populations from Nanma and Hengdian of Dongyang county, both of which import logs for producing furniture. These ants were collected from sites where imported logs were piled. (2) Two populations from Jinhua city: one from Luodian town of Wucheng district, a tree nursery center. This site was the first *S. invicta* detected in Zhejiang (2016), and the origin is believed to be southern China. The other population was collected from 20 sites distributed in Wucheng and Jindong districts, where the ants were likely spread from Luodian via tree transportation and nuptial flight. (3) Four populations from the other cities of Zhejiang, including Hangzhou, Wenzhou, Jiaxing, and Ningbo, which are located in different parts of the province. (4) The remaining 12 populations were collected from seven other provinces in eastern, southern, southwestern, and central China.

For most populations, three nests were sampled, spaced 100–500 m apart depending on nest availability. One population in Jinhua (JinH-2, Table 1) was sampled from 20 nests because fire ants in this district have a wide distribution range for the reasons described above. After gently disturbing each mound with a wooden stick, ants were collected into plastic bottles. We did not determine the social form (monogyne vs. polygyne) of the sampled colonies, as this study focused on broad-scale genetic structure. However, social form is known to influence genetic diversity and dispersal [13,14], and its absence is a limitation of our study. In the laboratory, ants were preserved in absolute ethanol and stored at −20 °C for subsequent DNA extraction.

It should be noted that the sampling effort was uneven across Zhejiang, with 17 of the 23 Zhejiang populations (74%) concentrated in Dongyang county, where imported log piles were the primary sampling sites. This overrepresentation of Dongyang may influence the Zhejiang-wide diversity indices and potentially accentuate the apparent homogeneity of the Dongyang cluster.

### 2.2. DNA Extraction and Microsatellite Amplification

One worker ant was randomly chosen from each sample. Total genomic DNA was extracted using the SteadyPure Universal Genomic DNA Extraction Kit (Accurate Biotechnology (Hunan) Co., Ltd., Changsha, China) following the manufacturer’s instructions. DNA concentration and purity were assessed to ensure accuracy of subsequent experiments.

A total of 66 microsatellite loci were amplified using the extracted DNA as template and primer pairs reported in the literature [12]. In each primer pair, one primer was labeled with a fluorescent dye (HEX, FAM, TET, or VIC) at the 5′ end. Multiple PCR amplifications were performed by combining two different primer pairs per reaction. The PCR reaction system consisted of 12.5 μL GoTaq^®^ Green Master Mix (2×) (Promega Corporation, Madison, WI, USA), 0.5 μL of each forward and reverse primer, 1.0 μL of DNA template, and 9.5 μL of ddH_2_O. Detailed information on primer sequences, PCR conditions, and thermal cycling profiles was adopted from previous studies [11,12,34]. PCR products were preliminarily detected by 2% agarose gel electrophoresis at 120 V for 30 min. Qualified products were sent to Hangzhou Baosirui Biotechnology Co., Ltd. (Hangzhou, China) for fluorescent capillary electrophoresis and microsatellite genotyping.

### 2.3. Microsatellite Data Analysis

Raw microsatellite data were converted into compatible formats using GenAlEx (v. 6.503) [35]. We calculated the proportion of missing data per locus to determine whether any loci should be excluded. Eight loci with a missing data proportion > 3.0% (Sdag C204, Sdag C278, seastones, Slipknot2, Sol-18, Solil 06, Solil 36 and Sunrise) were discarded. We noted that the exclusion of these eight loci does not affect the results or conclusions of the study. Null allele frequency for each locus in each population was estimated using FreeNA (v. 1.0) [36] using the expectation maximization algorithm described by Dempster et al. [37]. This analysis was performed to assess the suitability of the microsatellite markers, as null alleles arising from mutations in the primer binding site (or other unknown reasons) can prevent PCR amplification of the targeted locus and potentially bias the results.

### 2.4. Genetic Diversity of S. invicta Populations

Observed allele number (*N*_A_), effective allele number (*N*_E_), observed heterozygosity (*H*_O_), and expected heterozygosity (*H*_E_) for each locus across populations were estimated using POPGENE (v. 1.31; [38]). To assess overall genetic diversity in Zhejiang, we separately pooled data from the following groups: Dongyang populations (*n* = 51 individuals), Jinhua populations (*n* = 23), Zhejiang-wide populations (*n* = 84), and populations from the other seven provinces (*n* = 32). For each group, we estimated *N*_A_, *N*_E_, *H*_O_, *H*_E_, and *F*_IS_. Hardy–Weinberg equilibrium was tested for each group using GENEPOP (v. 4.8.3; [39]). Exact *p*-values were estimated using the Markov chain method (dememorization number = 10,000; number of batches = 20; iterations per batch = 5000). Based on the obtained *F*_IS_ and *p*-values, heterozygote excess or deficiency was assessed.

### 2.5. Population Differentiation

Genetic differentiation among the 35 populations was assessed using *F*_ST_. Pairwise *F*_ST_ values [40] were calculated with GENEPOP, and significance was tested with 10,000 permutations. Nei’s genetic distance (GD) was calculated using POPGENE. Principal co-ordinates analysis (PCoA) based on Nei’s genetic distance was conducted in GenAlEx to visualize clustering patterns among locations and individuals. To assess the potential impact of null alleles on *F*_ST_ estimates, we additionally computed pairwise *F*_ST_ values using the ENA (excluding null alleles) correction implemented in FreeNA [36]. Spearman’s rank correlation was used to compare the corrected and uncorrected pairwise *F*_ST_ matrices. Furthermore, PCoA based on the ENA corrected *F*_ST_ matrix was performed using the ape package (version 5.7-1) in R software (version 4.3.2), and data visualization was conducted using the ggplot2 package (version 4.0.2) [41], to visually evaluate whether the correction altered the overall clustering patterns.

Bayesian clustering analysis was performed using STRUCTURE (v. 2.3.4) [42] to infer genetic differentiation. An admixture ancestry model with correlated allele frequencies was applied. For each *K* (1 to 13), we ran 20 independent replicates, each consisting of 100,000 Markov chain Monte Carlo (MCMC) iterations after a burn-in of 50,000 iterations, with a thinning interval of 10. The optimal *K* was determined using the *ΔK* method [43] implemented in Structure Harvester [44] (https://lmme.ac.cn/StructureSelector/, accessed on 1 May 2026). Results from the 20 replicates for the best *K* were combined using CLUMPP (v. 1.1.2; [45]).

### 2.6. Statistical Analyses

To compare the genetic diversity indices (*N*_A_, *N*_E_, *H*_O_, *H*_E_) among the four groups (Dongyang, Jinhua, Zhejiang-wide, and other provinces), we first tested the data for normality using the Shapiro–Wilk test and homogeneity of variances using Levene‘s test. Shapiro–Wilk tests revealed that *N*_A_, *N*_E_ and *H*_E_ values were not normally distributed (*p* < 0.05), while *H*_O_ met the normality assumption (*p* > 0.05). Nevertheless, given the overall violation of normality for most indices and the small sample sizes, we employed nonparametric Kruskal–Wallis tests (significance level = 0.05) for all four indices for consistency. When a significant difference was detected, post hoc pairwise comparisons were performed using Dunn’s test. The resulting *p* values were adjusted with the Bonferroni correction, and differences were considered statistically significant if they fell below the corrected threshold corresponding to an overall alpha level of 0.05. Statistical analyses were performed using IBM SPSS Statistics for Windows (version 26.0) [46].

## 3. Results

### 3.1. Microsatellite Data Analysis

We genotyped a total of 116 *S. invicta* individuals from 35 locations collected from Zhejiang and other seven provinces (Table 1; Figure 1). Among the 66 microsatellite loci analyzed, 26 loci showed missing data in 0.86% to 11.21% of individuals (Figure S1). Eight loci, i.e., Sdag C204, Sdag C278, seastones, Slipknot2, Sol-18, Solil 06, Solil 36 and Sunrise, had missing data >3% due to amplification failure and were therefore excluded from the study. Preliminary analysis indicated that absence of these eight loci did not affect the results or conclusions. Of the remaining 58 loci, 18 loci (10.3%) were not successfully typed in only 0.86–2.59% of individuals. However, for 16 of these 58 loci, GENEPOP analysis revealed a risk for null alleles in more than 20% of locations. To reduce this risk, we discarded these markers, retaining 42 loci for further analysis.

### 3.2. Genetic Diversity of S. invicta Populations

Analysis of 42 microsatellite loci in 35 *S. invicta* populations revealed a total of 377 alleles, with the number of alleles per locus ranging from three (dire_wolf and Solil_10) to 19 (Sol-42f). Observed heterozygosity (*H*_O_) ranged from 0.0000 (Sinv-12, dire_wolf, and Sol i110) to 1.0000 (Bertha), with an average of 0.378, and *H*_E_ ranged from 0.0427 (jack_a_roe) to 0.8746 (Sdag C367), with an average of 0.534 (Table S1). These results indicate that the loci used in this study provide substantial genetic information, revealing high allelic variation and genetic diversity within *S. invicta* populations in China.

As considering the situation in four groups, Dongyang, Jinhua, Zhejiang-wide and other provinces, the Kruskal–Wallis test revealed a statistically significant difference among the groups in *N*_A_ (*H* = 33.790, *p* < 0.001), *N*_E_ (*H* = 2.235, *p* = 0.046) and *H*_E_ (*H* = 2.651, *p* = 0.049), but not in *H*_O_ (*H* = 0.099, *p* = 0.960). The Dongyang and Jinhua groups had significantly lower *N*_A_ (3.93 and 3.67 on average, respectively), *N*_E_ (2.27 and 2.21, respectively) and *H*_E_ (0.435 and 0.428, respectively) than the group of other provinces (6.52, 2.88 and 0.569, respectively; Table 2). None of these parameters differed significantly between the Zhejiang-wide and other-provinces group. The Hardy–Weinberg exact test showed that each group deviated significantly from equilibrium (*p* < 0.05). The Zhejiang-wide and other-provinces groups exhibited a slight heterozygote excess (*F*_IS_ < 0), likely due to admixture between individuals from different genetic backgrounds. The Dongyang group also showed a heterozygote excess (*F*_IS_ = −0.0721). This is unexpected if Dongyang populations represent a single, homogeneous overseas introduction (which would typically result in heterozygote deficiency or equilibrium). Instead, this excess suggests that even within the log pile sites of Dongyang, admixture among multiple, subtly differentiated source populations may be occurring. This could reflect the accumulation of ants from imported logs originating from different overseas locations or multiple introduction events over time. The Jinhua group showed a small but significant heterozygote deficiency (*F*_IS_ = 0.0675).

### 3.3. Genetic Differentiation of S. invicta Populations

Pairwise *F*_ST_ values between populations ranged from 0.0000 to 0.7632 (Table S2, below diagonal), with the largest values observed between Ningbo, Longyan and Huizhou (>0.7). Among the 23 Zhejiang populations, more than half (52.2%) had a mean *F*_ST_ value ≥ 0.25, each of them had a mean *F*_ST_ value of 0.18–0.46 (average 0.26), when compared with populations from other provinces. Pairwise *F*_ST_ values among Zhejiang populations ranged from 0.10 to 0.52. These results indicate that some populations in Zhejiang are highly differentiated and genetically distinct from those in other provinces.

**Figure 2:**
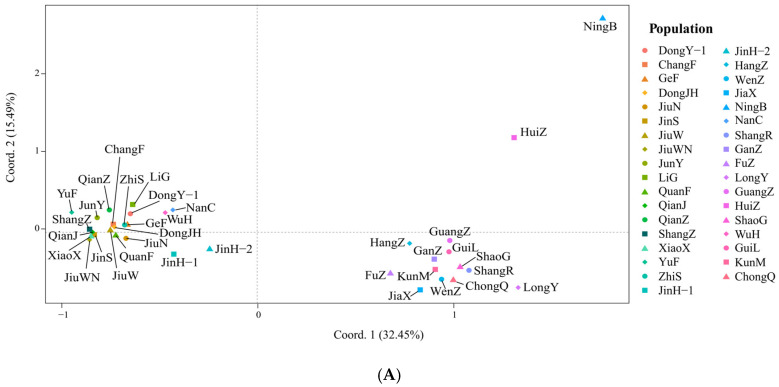

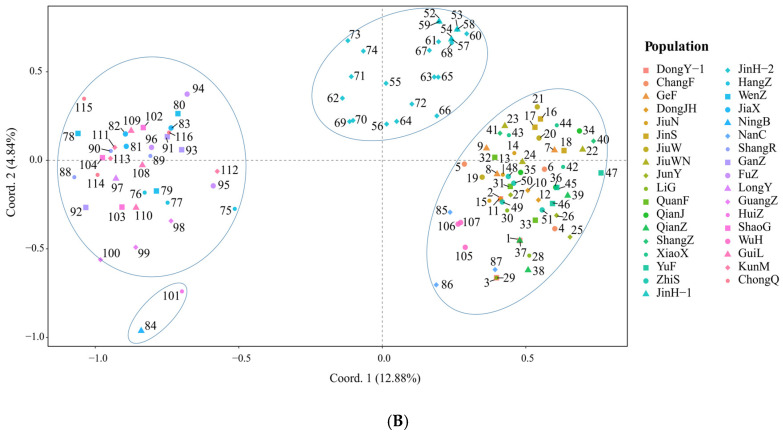
Principal coordinate analysis (PCoA) for 35 populations (**A**) and 116 individuals (**B**) based on 42 microsatellite loci. Refer to Table 1 for population IDs.

Principal coordinates analysis (PCoA) based on pairwise Nei’s genetic distances was performed, and the ordination of populations according to the first two axes is shown in Figure 2. The first two axes accounted for nearly 48% of the total genetic variance and revealed three distinct clusters (Figure 2A). Dongyang, Jinhua, Nanchang and Wuhan populations formed one cluster. Within this cluster, most Dongyang samples plotted closely together and were clearly separated from the two Jinhua populations, while Nanchang and Wuhan populations nearly overlapped. The second cluster included all other populations except Ningbo and Huizhou, namely Hangzhou, Jiaxing, and Wenzhou (Zhejiang) together with populations from Jiangxi, Fujian, Guangdong, Guangxi, Yunnan, and Chongqing. Ningbo and Huizhou formed the third, looser cluster, both strongly separated from the other populations. Individual-based PCoA yielded results similar to those of location-based PCoA (Figure 2B).

Comparison of pairwise *F*_ST_ values before and after ENA correction revealed an extremely high Spearman rank correlation (ρ = 0.996, *p* < 0.001), indicating that null alleles did not introduce substantial bias. PCoA based on the ENA corrected *F*_ST_ matrix (Figure S2) yielded clustering patterns that were largely identical to those from the uncorrected analysis (Figure 2A). A minor difference was observed: the Jiaxing (Zhejiang) and Longyan (Fujian) populations shifted from the second cluster to a more peripheral position. Nevertheless, all key differentiation patterns, particularly the distinctness of the Ningbo population and the clear genetic separation between Jinhua and Dongyang, remained unchanged. Therefore, null alleles did not materially affect our conclusions.

**Figure 3:**
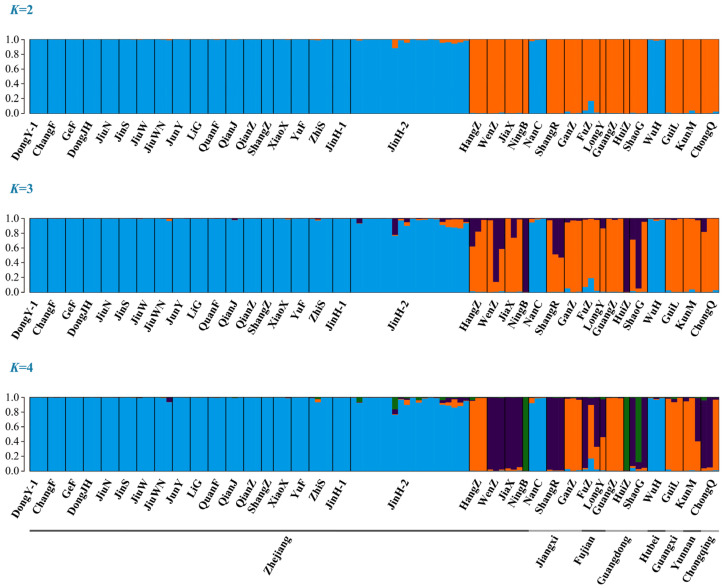
Population genetic structure analysis estimated by Bayesian simulation implemented in STRUCTURE using 42 microsatellite loci at *K* = 2, 3, and 4. Each individual is shown as a vertical bar representing ancestry.

Bayesian clustering with STRUCTURE, analyzed according to Evanno et al. [42], suggested *K* = 2 as the optimal value (Figure S3). A clear separation was observed between samples from Dongyang, Jinhua, Nanchang, and Wuhan, and samples from (a) four locations in Zhejiang (Hangzhou, Wenzhou, Jiaxing, and Ningbo); (b) Two locations in Jiangxi (Shangrao and Ganzhou); and (c) locations in Fujian, Guangdong, Guangxi, Yunnan, and Chongqing (Figure 3).

To explore further substructure and among-location genetic variation, we also examined higher *K* values. At *K* = 4, Ningbo and Huizhou samples formed their own cluster, with membership probabilities for this cluster also present in a fraction of Shaoguan samples. At *K* = 5, Jinhua samples formed their own cluster. At *K* = 6, Jiaxing samples (Zhejiang) formed a distinct genetic cluster, with their membership probabilities also present in Fujian and Guangdong. At *K* = 7 and *K* = 8, evident differentiation could be detected in Jinhua samples, whereas Dongyang samples showed a pattern very similar to that observed at lower *K* values. At *K* = 8, Hangzhou samples partially formed a cluster, with their membership probabilities clearly present in Jiangxi, Guangdong and Guangxi. (Figure S4).

These results are largely consistent with PCoA. They reveal that populations in Zhejiang exhibit several distinct genetic clusters as primary classification units (e.g., Ningbo, Dongyang, and Jinhua), indicating a more intricate genetic structure compared with populations from other provinces.

## 4. Discussion

Microsatellite markers are considered more variable than other molecular methods (e.g., mtDNA-based approaches) for revealing genetic diversity scenarios [47,48]. Here, we used this tool to investigate the status of *Solenopsis invicta* in Zhejiang, one of the most seriously invaded regions in China. Our PCoA and STRUCTURE analyses indicate that *S. invicta* populations in China are genetically differentiated, which is consistent with the findings of Yang et al. [14].

This suggests that fire ants in Ningbo may have a different source of introduction. A plausible hypothesis is an overseas origin via the port, as Ningbo has a major international port and fire ants are known to be introduced through this pathway [49,50,51,52]. However, we emphasize that this interpretation is based solely on negative evidence, i.e., the high differentiation from all domestic populations sampled. No overseas reference populations (e.g., from Southeast Asia, the Americas, or Taiwan, China) were included in this study. Therefore, while the overseas origin hypothesis is consistent with our data, it cannot be definitively confirmed. An alternative explanation that Ningbo represents a previously unsampled or genetically distinct domestic source cannot be ruled out. Future studies incorporating international reference samples are essential to test this hypothesis.

Clear genetic differentiation exists between the fire ant populations in Jinhua and Dongyang, even though the two locations are only ~55 km apart. This corroborates our speculation. Jinhua has numerous tree nurseries, and according to Huang et al. [31], the fire ants there probably originated from southern China, introduced through the frequent transportation of seedlings and mature trees. In contrast, Dongyang serves as one of the major wooden-furniture production bases in Zhejiang, where ants were likely introduced via imported logs from overseas. According to Wang et al. [53], among goods imported into China, logs are one of the dominant vectors carrying fire ants as hitchhikers. This scenario is plausible because all of our Dongyang samples were collected at sites where imported logs were piled. Interestingly, the observed heterozygote excess in Dongyang (*F*_IS_ = −0.0721) contradicts the expectation of a single, genetically depauperate introduction event. Instead, it suggests that multiple, genetically distinct fire ant propagules have arrived via different log shipments, likely from various overseas sources, and have subsequently admixed. This admixture could generate heterozygote excess and increase genetic diversity, potentially enhancing the invasive potential of these populations. Thus, the genetic signature of Dongyang is consistent with a “multiple overseas introductions” model rather than a single source. Therefore, the fire ant populations in Jinhua and Dongyang likely have different introduction sources. However, the high number of Dongyang samples (17 populations) relative to other Zhejiang locations should be considered; while this intensive sampling reveals local differentiation, it may also exaggerate the genetic distinctiveness of Dongyang compared to undersampled areas. Future sampling with more balanced geographic coverage across Zhejiang would help validate the degree of differentiation we observed.

To our surprise, the ants from the other three Zhejiang locations (Wenzhou, Jiaxing, and Hangzhou) are genetically more distant from Ningbo, Jinhua, and Dongyang (within the same province) than from locations in other provinces. Thus, fire ants in these three locations appear to originate from other Chinese provinces. Taken together, fire ants in some parts of Zhejiang were introduced through a long-distance jumping pattern, as previously described for this ant [14], indicating multiple invasion sources within this province.

Our study enriches the understanding of the overall genetic diversity of fire ants in China. The average expected heterozygosity (*H*_E_) of the sampled *S. invicta* populations was 0.534, ranging from 0.0427 to 0.8746 (Table S1), indicating a moderate level of genetic variation in this country. These *H*_E_ values show a wider range than those reported by Yang et al. [14] (*H*_E_ = 0.409–0.491), Huang et al. [25] (0.1705–0.3199, mean 0.2708), and Xiao et al. [16] (0.251–0.735, mean 0.471). Hence, the detected genetic variation may differ greatly among studies, depending on which locations (populations) were selected. Furthermore, our study suggests that even within a narrow geographical range (e.g., a single province), there may be weak gene flow of fire ants, as indicated by the pronounced among-location genetic differentiation in Zhejiang described above. Strong genetic structuring and weak gene flow in fire ants have been documented previously (e.g., [13]).

In the context of fire ant management in Zhejiang (and other coastal provinces), our study highlights the importance of reinforcing quarantine measures against red imported fire ants at ports (e.g., Ningbo). This is necessary not only because ants may be introduced through ports, but also because such introduced ants could admix with local populations, thereby increasing their genetic diversity. Increased genetic diversity can enhance fire ants’ ability to adapt to newly invaded environments and facilitate their establishment [54,55]. Moreover, management strategies should aim to reduce admixture among genetically distinct populations within the province, for example, by limiting the transport of nursery stocks between different regions. In addition to Ningbo, specific attention should be paid to other regions of this province. For instance, Jinhua, as a major hub for horticultural sapling transportation, exhibited a distinct genetic cluster in this study. Given its central location and high infestation level, Jinhua could act as a bridgehead for secondary spread to northern regions (e.g., Shanghai and Jiangsu). Future surveillance should prioritize monitoring sapling shipments originating from Jinhua.

It is worth noting that, in the present study, the majority of populations were represented by only three nests, and a few populations (e.g., Ningbo, ShangZ, Longyan, and Huizhou) were represented by only one or two nests (Table 1). Such limited sampling sizes may cause potential bias in genetic diversity estimation, reduce the probability of detecting rare alleles, and raise concerns regarding the robustness of the inferred genetic differentiation, particularly for those populations with minimal sample sizes. Nevertheless, the analytical methods employed, principal coordinates analysis (PCoA) and Bayesian clustering as implemented in STRUCTURE, are well recognized for their efficacy in detecting and resolving population genetic structure even with modest sample sizes [42,43]. PCoA transforms multilocus genetic distances into orthogonal axes, enabling clear visual discrimination of individuals based on overall genetic similarity; this approach is generally robust to unequal sample sizes when underlying differentiation is pronounced. STRUCTURE adopts a model-based probabilistic framework to assign individuals to genetic clusters, and previous simulation studies have demonstrated its reliability in recovering true population structure with as few as one or two individuals per population, provided that the markers are sufficiently informative and the differentiation is substantial [42,43]. In the current dataset, populations exhibiting strong differentiation, such as Ningbo, Jinhua, and Dongyang, consistently emerged as distinct clusters across both PCoA and STRUCTURE analyses. These patterns remained stable under varying *K* values and across independent runs. Therefore, the limited sample sizes in a few populations do not compromise the principal conclusions of this study concerning the multiple introduction sources and the genetic structuring of *S. invicta* in Zhejiang. However, we do acknowledge that this limitation primarily affects the reliability of quantitative metrics such as allele frequencies, expected heterozygosity, and exact *F*_ST_ values reported in Table 2 and Table S2, which should be interpreted with caution and may be subject to change with larger samples.

A caveat of our study is that we did not assess the social form (monogyne or polygyne) of the sampled colonies. The two social forms of *S. invicta* exhibit profound differences in colony structure, dispersal behavior, and population genetics [14]. Polygyne colonies, for example, are often less genetically diverse and can show weaker population structuring due to higher queen densities and local dispersal [56]. The strong differentiation we observed in Zhejiang (e.g., between Jinhua and Dongyang) might be partially influenced by differences in social form, although the geographic proximity and distinct introduction pathways we propose remain plausible. Future studies should combine microsatellite genotyping with social form diagnostics (e.g., *Gp-9* genotyping [57]) to disentangle the effects of introduction history and social structure.

Finally, we hope our study serves as an example of multiple invasion sources of fire ants in eastern China, a pattern also observed in Fujian, another province in the region [32]. Further monitoring of fire ant genetic variation in this region is needed, as it can help identify reinvasion sources, understand spread dynamics, and evaluate the effectiveness of control interventions, as suggested by Rollins et al. [58] and Destour et al. [59].

## 5. Conclusions

Understanding the introduction sources of invasive species is critical for predicting their spread and designing effective management strategies. For the red imported fire ant in eastern China, previous work based on mitochondrial DNA suggested a single or limited origin from southern China, overlooking the possibility of multiple, concurrent introductions via diverse human mediated vectors. In this study, we used 42 microsatellite loci to resolve the genetic structure of *S. invicta* across Zhejiang Province and seven other Chinese provinces. Our results reveal a far more complex invasion history than previously recognized. The Ningbo population is genetically highly distinct from all other sampled Chinese populations, providing evidence consistent with an overseas introduction (e.g., via port entry)—a scenario not considered before. Despite being only ~55 km apart, the Jinhua and Dongyang populations are clearly differentiated, reflecting separate introduction pathways linked to nursery plant transport and imported logs, respectively. Moreover, populations in Wenzhou, Jiaxing and Hangzhou are genetically closer to extra-provincial samples than to other Zhejiang populations, indicating additional domestic sources. Collectively, these findings demonstrate that the invasion of *S. invicta* in Zhejiang has resulted from multiple independent introduction events involving both domestic and international sources. This study advances invasion biology by showing that even at a provincial scale, genetically distinct propagules can accumulate and admix, potentially enhancing adaptive potential. It also provides directly actionable insights: region-specific quarantine inspections (especially at Ningbo port), stricter regulation of nursery stock and log movements, and the need for ongoing genetic monitoring to detect new introductions and prevent admixture that could exacerbate invasiveness.

## Figures and Tables

**Table 2:** Indices of genetic diversity in populations of four groups.

Group	*N*_A_	*N*_E_	*H*_O_	*H*_E_	*F*_IS_
Dongyang group	3.93 ± 2.29 b	2.27 ± 1.19 b	0.350 ± 0.269 a	0.435 ± 0.286 b	−0.0721
Jinhua group	3.67 ± 2.13 b	2.21 ± 1.14 b	0.350 ± 0.299 a	0.428 ± 0.284 b	0.0675
Zhejiang-wide group	6.64 ± 3.43 a	2.49 ± 1.31 ab	0.357 ± 0.265 a	0.487 ± 0.255 ab	−0.1031
Other provinces group	6.52 ± 3.14 a	2.88 ± 1.57 a	0.378 ± 0.256 a	0.569 ± 0.205 a	−0.0829

Note: *N*_A_, mean alleles number per locus; *N*_E_, mean effective alleles number per locus; *H*_O_, observed heterozygosity; *H*_E_, expected heterozygosity; *F*_IS_, inbreeding coefficient within population. Data are in mean ± SD. Kruskal–Wallis test was used, followed by Dunn’s test with Bonferroni correction. Values in the same column with the same lowercase letter do not differ significantly among groups (adjusted *p* > 0.05).

## Data Availability

The original contributions presented in this study are included in the article/Supplementary Material. Further inquiries can be directed to the corresponding authors.

## Supplementary Materials

The following supporting information can be downloaded at: https://www.mdpi.com/article/10.3390/insects17060597/s1, Figure S1: Percentage of missing data over the localities in the locus dataset; Table S1: Indices of genetic diversity per locus deduced across 35 populations; Table S2: Pairwise *F*_ST_ values (below diagonal) and Nei’s unbiased genetic distance (above diagonal) between 35 populations based on 42 microsatellite loci; Figure S2: PCoA for 35 populations based on pairwise *F*_ST_ values computed using the ENA correction method implemented in FreeNA. Refer to Table 1 for population IDs; Figure S3: Estimation of the number of clusters (*K*) based on 20 runs of ranging from 1 to 13 using STRUCTURE 2.3.4; Figure S4: Population genetic structure analysis estimated by Bayesian simulation implemented in STRUCTURE using 42 microsatellite loci at *K* = 5, 6, 7, and 8.

## References

[1] Hulme P.E. (2009). Trade, transport and trouble: Managing invasive species pathways in an era of globalization. J. Appl. Ecol..

[2] Chown S.L., Hodgins K.A., Griffin P.C., Oakeshott J.G., Byrne M., Hoffmann A.A. (2014). Biological invasions, climate change and genomics. Evol. Appl..

[3] Bradshaw C.J.A., Leroy B., Bellard C., Roiz D., Albert C., Fournier A., Barbet-Massin M., Salles J.M., Simard F., Courchamp F. (2016). Massive yet grossly underestimated global costs of invasive insects. Nat. Commun..

[4] Doherty T.S., Glen A.S., Nimmo D.G., Ritchie E.G., Dickman C.R. (2016). Invasive predators and global biodiversity loss. Proc. Natl. Acad. Sci. USA.

[5] David P., Thébault E., Anneville O., Duyck P.F., Chapuis E., Loeuille N., Bohan D.A., Dumbrell A.J., Massol F. (2017). Impacts of invasive species on food webs. Networks of Invasion: A Synthesis of Concepts.

[6] Foucaud J., Orivel J., Loiseau A., Delabie J.H.C., Jourdan H., Konghouleux D., Vonshak M., Tindo M., Mercier J.L., Fresneau D. (2010). Worldwide invasion by the little fire ant: Routes of introduction and eco-evolutionary pathways. Evol. Appl..

[7] Loiseau A., Kergoat G.J., Blight O., Demetriou J., Espadaler X., Benoit L., Calcaterra L.A., Chifflet L., Jourdan H., Menchetti M. (2025). Newcomers and old friends: Long-distance and bridgehead introductions both contribute to the recent invasion of the little fire ant in southern Europe. Divers. Distrib..

[8] Ross K.G., Shoemaker D.D., Krieger M.J.B., DeHeer C.J., Keller L. (1999). Assessing genetic structure with multiple classes of molecular markers: A case study involving the introduced fire ant *Solenopsis invicta*. Mol. Biol. Evol..

[9] Zima J., Lebrasseur O., Borovanská M., Janda M. (2016). Identification of microsatellite markers for a worldwide distributed, highly invasive ant species *Tapinoma melanocephalum* (Hymenoptera: Formicidae). Eur. J. Entomol..

[10] Henshaw M.T., Kunzmann N., Vanderwoude C., Sanetra M., Crozier R.H. (2005). Population genetics and history of the introduced fire ant, *Solenopsis invicta* Buren (Hymenoptera: Formicidae), in Australia. Aust. J. Entomol..

[11] Ascunce M.S., Bouwma A.M., Shoemaker D. (2009). Characterization of 24 microsatellite markers in 11 species of fire ants in the genus *Solenopsis* (Hymenoptera: Formicidae). Mol. Ecol. Resour..

[12] Ascunce M.S., Yang C.C., Oakey J., Calcaterra L., Wu W.J., Shih C.J., Goudet J., Ross K.G., Shoemaker D. (2011). Global invasion history of the fire ant *Solenopsis invicta*. Science.

[13] Yang C.C., Shoemaker D.D., Wu W.J., Shih C.J. (2008). Population genetic structure of the red imported fire ant, *Solenopsis invicta*, in Taiwan. Insects Soc..

[14] Yang C.C., Ascunce M.S., Luo L.Z., Shao J.G., Shih C.J., Shoemaker D. (2012). Propagule pressure and colony social organization are associated with the successful invasion and rapid range expansion of fire ants in China. Mol. Ecol..

[15] Matheny A.M., Kimmel L.B., Stone P.A., Fenwick A.M. (2018). Comparative population genetics of red imported fire ants (*Solenopsis invicta*) at the University of Central Oklahoma and Lake Arcadia, Edmond, Oklahoma. Am. Midl. Nat..

[16] Xiao Q., Wang L., Chen S.Q., Zheng C.Y., Lu Y.Y., Xu Y.J. (2023). Gut microbiome composition of the fire ant *Solenopsis invicta*: An integrated analysis of host genotype and geographical distribution. Microbiol. Spectr..

[17] Lowe S., Browne M., Boudjelas S., De Poorter M. (2004). 100 of the World’s Worst Invasive Alien Species: A Selection from the Global Invasive Species Database.

[18] Menchetti M., Schifani E., Alicata A., Cardador L., Sbrega E., Toro-Delgado E., Vila R. (2023). The invasive ant *Solenopsis invicta* is established in Europe. Curr. Biol..

[19] (2026). EPPO. EPPO Global Database. https://gd.eppo.int/taxon/SOLEIN.

[20] Wang L., Chen K.W., Feng X.D., Wang X.L., Lu Y.Y. (2022). Long-term predication of red imported fire ant (*Solenopsis invicta* Buren) expansion in mainland China. J. Environ. Entomol..

[21] Ministry of Agriculture and Rural Affairs of PRC (2025). List of Administrative Regions for the Distribution of Agricultural Plant Quarantine Pests in China (as of 30 June 2025). https://zzys.moa.gov.cn/tzgg/202507/t20250730_6476125.htm.

[22] Song J., Zhang H., Li M., Han W., Yin Y., Lei J. (2021). Prediction of Spatiotemporal Invasive Risk of the Red Import Fire Ant, *Solenopsis invicta* (Hymenoptera: Formicidae), in China. Insects.

[23] Xu Y.L., Qing Y.J., Liu H., Zhu J.Q., Li Z.H., Fang Y., Ma C., Zhao S.Q. (2022). Suitability analysis of *Solenopsis invicta* considering the factor of agricultural protected areas. J. Environ. Entomol..

[24] Li M., Zhao H., Xian X., Zhu J., Chen B., Jia T., Wang R., Liu W. (2023). Geographical distribution pattern and ecological niche of *Solenopsis invicta* Buren in China under climate change. Diversity.

[25] Huang Y.W., He X.F., Lu Y.Y., Zeng L., Cheng D.F. (2014). Population genetic structure of *Solenopsis invicta* Buren in China based on microsatellite. J. Biosaf..

[26] Zhang T.Y., Li X.Y., Gong C.W., Wang Y.M., Wen Y., Zhu X.C., Li B., Wang X.G. (2022). Investigation on the distribution and genetic structure of *Solenopsis invicta* Buren in Sichuan Province. J. Sichuan Agric. Univ..

[27] Ni M.H., Jiang M.X. (2025). Temporal dynamics of colony relocation in the red imported fire ant, *Solenopsis invicta*, and its associations with habitat type and social form. Acta Ecol. Sin..

[28] Ni M.H., Lu J.L., Yang X.Y., Zheng Y.R., Wang Y., Jiang M.X. (2025). Underground inter-nest tunnels of red imported fire ants, *Solenopsis invicta*: Physical features, and associations with colony and environmental factors. Insects.

[29] Ni M.H., Yang X.Y., Zheng Y.R., Wang Y., Jiang M.X. (2024). Discovering native ant species with the potential to suppress red imported fire ants. Insects.

[30] Ni M.H., Yang X.Y., Qian M.H., Jiang M.X. (2025). Blocking effects of landscape factors against dispersal of the red imported fire ant, *Solenopsis invicta*. J. Environ. Entomol..

[31] Huang J., Yu C.D., Wang S.Z., Bao J.D., Jiang Z.T., Dong W.Y., Su H.L., Chen L.M., Ullah F., Zhou S.X. (2025). Genetic structure, dispersal pathways, and northern expansion predictions of *Solenopsis invicta*: A two-decade journey through China’s diverse landscapes. Insect Sci..

[32] Zhang X., Hou Y.M. (2014). Invasion history of *Solenopsis invicta* (Hymenoptera: Formicidae) in Fujian, China based on mitochondrial DNA and its implications in development of a control strategy. Insect Sci..

[33] Ricciardi A., Blackburn T.M., Carlton J.T., Dick J.T.A., Hulme P.E., Iacarella J.C., Jeschke J.M., Liebhold A.M., Lockwood J.L., MacIsaac H.J. (2017). Invasion science: A horizon scan of emerging challenges and opportunities. Trends Ecol. Evol..

[34] Ross K.G., Gotzek D., Ascunce M.S., Shoemaker D.D. (2010). Species delimitation: A case study in a problematic ant taxon. Syst. Biol..

[35] Peakall R.O.D., Smouse P.E. (2005). GENALEX 6: Genetic analysis in Excel. Population genetic software for teaching and research. Mol. Ecol. Notes.

[36] Chapuis M.P., Estoup A. (2007). Microsatellite null alleles and estimation of population differentiation. Mol. Biol. Evol..

[37] Dempster A.P., Laird N.M., Rubin D.B. (1977). Maximum likelihood from incomplete data via the EM algorithm. J. R. Stat. Soc. Ser. B.

[38] Yeh F.C., Yang R.C., Boyle T. (1999). POPGENE.

[39] Raymond M., Rousset F. (1995). GENEPOP (Version 1.2): Population genetics software for exact tests and ecumenicism. J. Hered..

[40] Weir B.S., Cockerham C.C. (1984). Estimating F-statistics for the analysis of population structure. Evolution.

[41] Wickham H. (2016). ggplot2: Elegant Graphics for Data Analysis.

[42] Pritchard J.K., Stephens M., Donnelly P. (2000). Inference of population structure using multilocus genotype data. Genetics.

[43] Evanno G., Regnaut S., Goudet J. (2005). Detecting the number of clusters of individuals using the software structure: A simulation study. Mol. Ecol..

[44] Earl D.A., vonHoldt B.M. (2012). STRUCTURE HARVESTER: A website and program for visualizing STRUCTURE output and implementing the Evanno method. Conserv. Genet. Resour..

[45] Jakobsson M., Rosenberg N.A. (2007). CLUMPP: A cluster matching and permutation program for dealing with label switching and multimodality in analysis of population structure. Bioinformatics.

[46] IBM Corp. (2019). IBM SPSS Statistics for Windows.

[47] Cesari M., Maistrello L., Piemontese L., Bonini R., Dioli P., Lee W., Park C.G., Partsinevelos G.K., Rebecchi L., Guidetti R. (2018). Genetic diversity of the brown marmorated stink bug *Halyomorpha halys* in the invaded territories of Europe and its patterns of diffusion in Italy. Biol. Invasions.

[48] Machado D.D., Costa E.C., Guedes J.V.C., Barbosa L.R., Martínez G., Mayorga S.I., Ramos S.O., Branco M., Garcia A., Vanegas-Rico J.M. (2020). One maternal lineage leads the expansion of *Thaumastocoris peregrinus* (Hemiptera: Thaumastocoridae) in the New and Old Worlds. Sci. Rep..

[49] Wylie R., Yang C.C.S., Tsuji K. (2019). Invader at the gate: The status of red imported fire ant in Australia and Asia. Ecol. Res..

[50] Li D.X., Li Z.X., Wang X.X., Wang L., Khoso A.G., Liu D.G. (2023). Climate change and international trade can exacerbate the invasion risk of the red imported fire ant *Solenopsis invicta* around the globe. Entomol. Gen..

[51] Homma S., Murakami D., Hosokawa S., Kanefuji K. (2025). Introduction risk of fire ants through container cargo in ports: Data integration approach considering a logistic network. PLoS ONE.

[52] Kim D., Lee H., Kim N., Jang B.J., Kim D.E. (2025). Monitoring of ant species surrounding the ports of South Korea. Biodivers. Data J..

[53] Wang L., Zeng L., Xu Y.J., Lu Y.Y. (2020). Prevalence and management of *Solenopsis invicta* in China. NeoBiota.

[54] Frankham R. (2004). Invasion biology—Resolving the genetic paradox in invasive species. Heredity.

[55] Garnas J.R., Auger-Rozenberg M.A., Roques A., Bertelsmeier C., Wingfield M.J., Saccaggi D.L., Roy H.E., Slippers B. (2016). Complex patterns of global spread in invasive insects: Eco-evolutionary and management consequences. Biol. Invasions.

[56] Shoemaker D.D., DeHeer C.J., Krieger M.J.B., Ross K.G. (2006). Population genetics of the invasive fire ant *Solenopsis invicta* (Hymenoptera: Formicidae) in the United States. Ann. Entomol. Soc. Am..

[57] Krieger M.J.B., Ross K.G. (2002). Identification of a major gene regulating complex social behavior. Science.

[58] Rollins L.A., Woolnough A.P., Wilton A.N., Sinclair R., Sherwin W.B. (2009). Invasive species can’t cover their tracks: Using microsatellites to assist management of starling (*Sturnus vulgaris*) populations in Western Australia. Mol. Ecol..

[59] Destour G., Kaufmann B., Centanni J., Abdelli Z., Doums C., Dumet A., Gippet J., Gomel L., Lucas A., Tauru H. (2025). Genetic tracing reveals the role of ornamental plant trade in the simultaneous spread of three invasive ant species in Western Europe. Peer Community J..

