# Supplementary material for: Genetic Structure Analyses of Red Imported Fire Ants in Zhejiang Reveal Multiple Introduction Sources in Eastern China
Source: Insects. 2026 Jun 7;17(6):597. doi: 10.3390/insects17060597 (PMC13300376; doi:10.3390/insects17060597)
Supplement: Supplementary file 1 [file insects-17-00597-s001.zip › insects-4323756-supplementary.pdf]

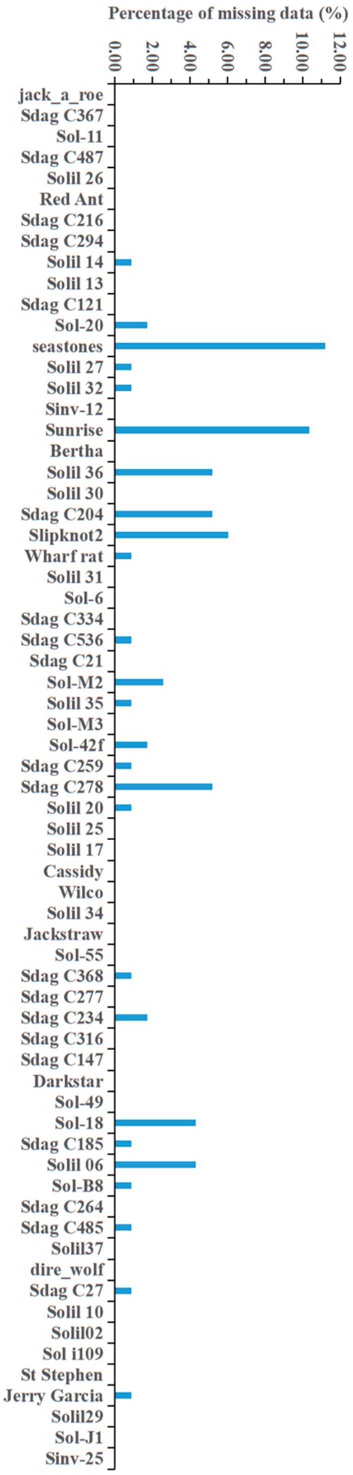

Figure S1. Percentage of missing data over the localities in the locus dataset.

Table S1. Indices of genetic diversity per locus deduced across 35 populations.

Locus	N_A	N_E	H_O	H_E
jack_a_roe	6	1.0444	0.0259	0.0427
Sdag C367	17	7.7422	0.8103	0.8746
Sol-11	14	7.0543	0.6466	0.8620
Solil 26	7	2.1332	0.5517	0.5335
Red Ant	4	1.4102	0.1897	0.2921
Sdag C216	8	4.5185	0.6034	0.7821
Solil 14	5	3.1948	0.5739	0.6900
Solil 13	14	4.0306	0.5086	0.7552
Sdag C121	10	2.7472	0.4138	0.6388
Solil 27	5	2.2863	0.4870	0.5651
Solil 32	5	2.1039	0.4522	0.5270
Sinv-12	4	1.0911	0.0000	0.0839
Bertha	11	4.6899	1.0000	0.7904
Solil 30	4	1.7182	0.0086	0.4198
Sdag C334	15	3.2495	0.2586	0.6953
Sdag C536	6	1.4161	0.0609	0.2951
Sdag C21	6	1.9228	0.2931	0.4820
Sol-M2	12	3.3763	0.5221	0.7069
Sol-42f	19	3.8128	0.6228	0.7410
Solil 20	8	1.8878	0.4000	0.4723
Solil 25	8	2.7498	0.7069	0.6391
Solil 17	10	1.2414	0.0603	0.1953
Wilco	5	1.3419	0.0086	0.2559
Solil 34	12	1.1423	0.0431	0.1251
Jackstraw	12	3.7840	0.4224	0.7389
Sol-55	18	5.0172	0.5603	0.8041
Sdag C368	7	2.0906	0.4435	0.5239
Sdag C277	5	2.0267	0.0086	0.5088
Sdag C234	10	4.1514	0.7368	0.7625
Sdag C316	8	2.1221	0.3793	0.5310
Sol-49	11	4.3959	0.6121	0.7759
Sdag C185	12	2.2640	0.8000	0.5607
Sol-B8	6	1.9440	0.3478	0.4877
Sdag C485	14	4.0228	0.5739	0.7547
Solil37	6	1.4771	0.0948	0.3244
dire_wolf	3	2.0345	0.0000	0.5107
Sdag C27	16	6.4591	0.6957	0.8489
Solil 10	3	1.0906	0.0000	0.0834
St Stephen	6	1.1310	0.0776	0.1164
Solil29	10	1.8837	0.1724	0.4712
Sol-J1	8	2.8227	0.3793	0.6485
Sinv-25	7	1.9629	0.3103	0.4927
Mean	8.98	2.82	0.378	0.534
Total	377			

Note: N_A, allele number; N_E, effective allele number; H_O, observed heterozygosity; H_E, expected heterozygosity.

Table S2. Pairwise F_{ST} values (below diagonal) and Nei's unbiased genetic distance (above diagonal) between 35 populations based on 42 microsatellite loci. The full table is provided in the Supplementary Materials.

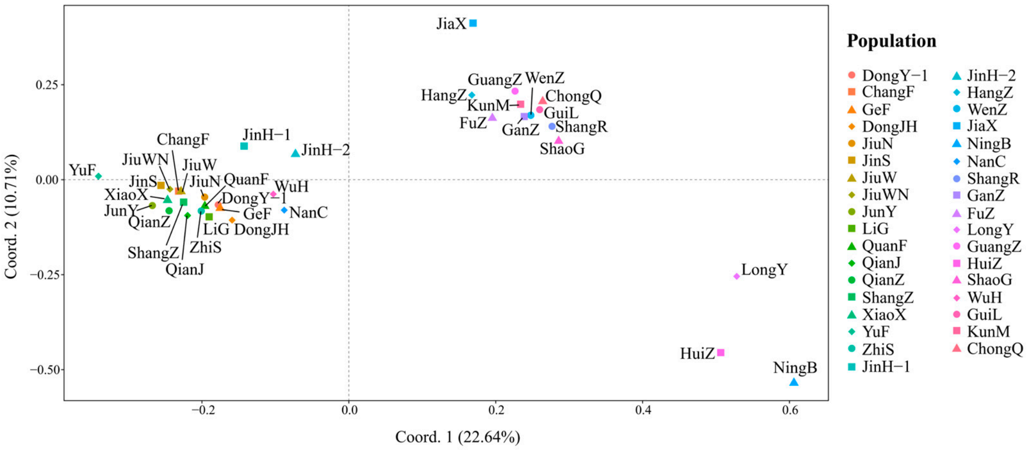

Figure S2. PCoA for 35 populations based on pairwise F_{ST} values computed using the ENA correction method implemented in FreeNA. Refer to Table 1 for population IDs.

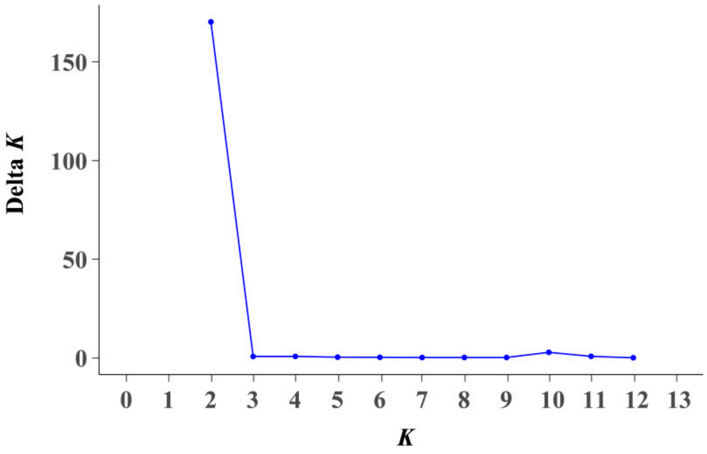

Figure S3. Estimation of the number of clusters (K) based on 20 runs of ranging from 1 to 13 using STRUCTURE 2.3.4.

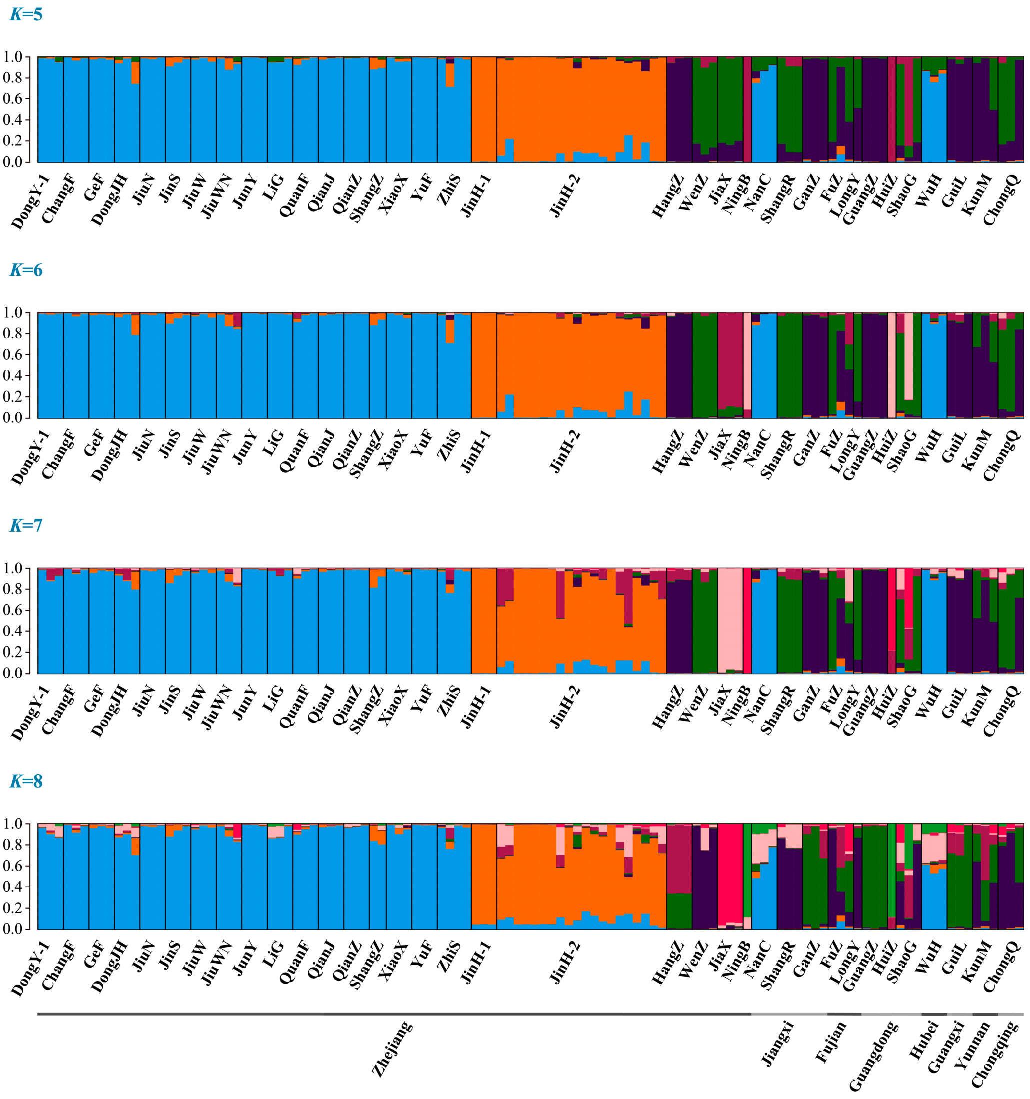

Figure S4. Population genetic structure analysis estimated by Bayesian simulation implemented in STRUCTURE using 42 microsatellite loci at $K = 5, 6, 7$, and 8.
